# Nonpathogenic leaf‐colonizing bacteria elicit pathogen‐like responses in a colonization density‐dependent manner

**DOI:** 10.1002/pei3.10137

**Published:** 2024-03-13

**Authors:** Moritz Miebach, Léa Faivre, Daniel Schubert, Paula Jameson, Mitja Remus‐Emsermann

**Affiliations:** ^1^ School of Biological Sciences University of Canterbury Christchurch New Zealand; ^2^ Biomolecular Interaction Centre University of Canterbury Christchurch New Zealand; ^3^ Department of Biology, Chemistry, Pharmacy, Institute of Biology ‐ Microbiology and Dahlem Centre of Plant Sciences] Freie Universität Berlin Berlin Germany

**Keywords:** *Arabidopsis*, chromatin state analysis, microbiota, phyllosphere, *Pseudomonas*, transcriptomics, *Williamsia*

## Abstract

Leaves are colonized by a complex mix of microbes, termed the leaf microbiota. Even though the leaf microbiota is increasingly recognized as an integral part of plant life and health, our understanding of its interactions with the plant host is still limited. Here, mature, axenically grown *Arabidopsis thaliana* plants were spray inoculated with six diverse leaf‐colonizing bacteria. The transcriptomic changes in leaves were tracked over time and significant changes in ethylene marker (*ARL2*) expression were observed only 2–4 days after spray inoculation. Whole‐transcriptome sequencing revealed that 4 days after inoculation, leaf transcriptional changes to colonization by nonpathogenic and pathogenic bacteria differed in strength but not in the type of response. Inoculation of plants with different densities of the nonpathogenic bacterium *Williamsia* sp. Leaf354 showed that high bacterial titers resulted in disease phenotypes and led to severe transcriptional reprogramming with a strong focus on plant defense. An in silico epigenetic analysis of the data was congruent with the transcriptomic analysis. These findings suggest (1) that plant responses are not rapid after spray inoculation, (2) that plant responses only differ in strength, and (3) that plants respond to high titers of nonpathogenic bacteria with pathogen‐like responses.

## Graphical abstract

*Arabidopsis* responds to colonization of high numbers of nonpathogenic bacteria with pathogen‐like transcriptomic responses. This is congruent with changes of the chromatin state of the treated plants.

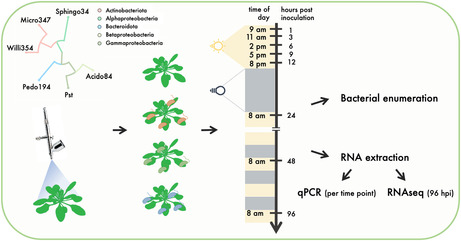

## 1 INTRODUCTION

Plants are colonized by a vast variety of bacteria with various effects on plant health and growth, ranging from pathogens to nitrogen‐fixing rhizobacteria (Kelly et al., 2018; Wang et al., 2018). Past research mainly focused on a few important microbiota members, overlooking most of the remarkably diverse microbes present on and within plants. Increasing evidence showcases the positive effects of the plant microbiota on its host, including the protection against abiotic (Lau & Lennon, 2012) and biotic (Innerebner et al., 2011; Ritpitakphong et al., 2016; Vogel et al., 2021; Zengerer et al., 2018) stressors, the promotion of growth (Spaepen et al., 2009), and the assimilation of specific nutrients (Hacquard et al., 2015; Singh et al., 2022). Some of these beneficial effects are solely attributed to microbes, whereas others involve the plant as an interaction partner. For example, with regard to the protection against biotic stress, some microbes can protect the plant from a pathogen via resource competition with the pathogen (Ji & Wilson, 2002) or the production of antimicrobials targeting the pathogen (Zengerer et al., 2018), whereas others stimulate the plant immune system, leading to increased immune responses upon subsequent infection with a pathogen (Pieterse et al., 1996; Vogel et al., 2016, 2021).

Even though the microbiota seems to be an integral part of plant life, our understanding of its interaction with the host is still limited. Studies on plant pathogens have shown that early perception of microbes is conferred by the detection of microbe‐associated molecular patterns (MAMPs). MAMPs are conserved traits of microbes irrespective of their symbiotic relationship with the plant, raising the question if and how plants discriminate between pathogenic and beneficial bacteria. Recently, it was shown that plants respond to diverse nonpathogenic leaf colonizers, at varying intensities of transcriptional reprogramming (Maier et al., 2021). However, whether the amount of transcriptional reprogramming is caused by the different identities of the bacteria is unclear. Notably, the observed plant response was enriched for plant defense‐associated genes and measured 9 days after inoculation of 10 days old seedlings, indicating persistently active immune responses. This is intriguing, since plant immune responses infer a growth penalty, commonly referred to as the growth–defense tradeoff (He et al., 2022; Huot et al., 2014).

As a persistent immune activation infers a growth penalty, plants need to activate their immune system according to potential pathogen threat in a timely manner (He et al., 2022; Huot et al., 2014). Different activation kinetics likely lead to different downstream signaling events and finally to a different immune response, highlighting the importance of time‐resolved analyses. In other words, MAMPs are present early in the plant–microbe interaction and lead to early plant responses. However, MAMPs are less indicative of the symbiotic relationship between the microbe and the plant compared to effector proteins. Accordingly, plant responses triggered by MAMPs are more transient than those triggered by effector molecules (Gao et al., 2013; Lamb & Dixon, 1997; Tsuda et al., 2013). Plant responses to different MAMPs are almost identical early after MAMP application, but differ later, resulting in differential immune outputs (Bjornson et al., 2021; Kim et al., 2014; Zipfel et al., 2006). Elf18 and chitosan, for example, predominantly activate jasmonic acid (JA)‐mediated immune responses early on, resulting in JA‐mediated immunity later, while flg22 activates jasmonic acid (JA)‐mediated and ethylene (ET)‐mediated immune responses early on, resulting in salicylic acid (SA)‐mediated immunity later (Kim et al., 2014). This further highlights the importance of time‐resolved analyses and suggests that, already at the level of MAMP recognition, plant immune responses differ depending on the cocktail of MAMPs present.

The response of plants to leaf colonization, whether by pathogenic or nonpathogenic bacteria, involves a significant transcriptome reprogramming (Maier et al., 2021; Moore et al., 2011). This reprogramming relies on the interaction of numerous transcription factors with the local chromatin environment. Indeed, several histone modifications such as H3K4me3, H3K36me3, and lysine acetylation were found to contribute to the induction of genes following pathogen exposure (Berr et al., 2012; Ding & Wang, 2015). By contrast, transcriptional reprogramming following colonization by nonpathogenic bacteria has not been investigated.

To monitor the dynamics of plant immune responses, we inoculated *Arabidopsis thaliana* (*Arabidopsis*) plants with diverse bacteria, including a plant pathogen, and measured transcriptional outputs, representing the three major phytohormone pathways in plant immunity (SA, JA, and ET), at various times after inoculation. As the strongest responses were observed 96 hours post inoculation (hpi), we measured the whole transcriptional plant responses at this time. Interestingly, plant responses to various bacterial inoculants strongly overlapped and we observed a trend that the plant responses were dependent on bacterial density. Most differentially expressed genes in response to one isolate were also differentially expressed in response to isolates that exhibited overall stronger responses. The question then arose whether nonpathogenic bacteria could induce plant defense responses at artificially high colonization densities. To address this question, we assessed the effects of different bacterial densities of the nonpathogenic leaf colonizer *Williamsia* sp. Leaf354 on *in planta* transcription as well as on plant health and plant weight 96 hpi and 21 dpi, respectively, to test the hypothesis that plants are monitoring bacterial population density rather than differentiating between different bacterial colonizers. Finally, in an effort to uncover chromatin marks that might contribute to transcriptional responses to nonpathogenic leaf colonizers, we performed an in silico chromatin analysis.

## 2 MATERIALS AND METHODS

### 2.1 Plant growth

*Arabidopsis* Col0 plants were grown as described previously (Miebach et al., 2020). Briefly, sterilized seeds were germinated on ½ MS (Murashige and Skoog medium, including vitamins, Duchefa, Haarlem, the Netherlands) 1% phytoagar (Duchefa) filled pipette tips. Healthy looking seedlings were aseptically transferred, without removal from the pipette tip, into Magenta boxes (Magenta vessel GA‐7, Magenta LLC, Lockport, IL, USA) filled with autoclaved ground Zeolite (sourced from cat litter—Vitapet, Purrfit Clay Litter, Masterpet New Zealand, Lower Hutt, New Zealand), and watered with 60 mL ½ MS. Each box received four seedlings. The boxes were closed with lids that allowed for gas exchange and placed into a climate cabinet (85% relative humidity, 11‐h light:13‐h dark cycle, 21°C, 150–200 μmol light intensity). Plants were grown for 4 weeks for the time course and 6 weeks for the bacterial density experiment before they were treated with bacteria or mock controls.

### 2.2 Plant inoculation

Bacterial suspensions were prepared as described previously (Miebach et al., 2020). Briefly, bacteria were cultivated at 30°C on minimal media agar plates containing 0.1% pyruvate as a carbon source. Bacterial suspensions were prepared from bacterial colonies suspended in phosphate‐buffered saline (PBS, 0.2 g L^−1^ NaCl, 1.44 g L^−1^ Na_2_HPO_4_, and 0.24 g L^−1^ KH_2_PO_4_) and washed twice via centrifugation at 4000×*g* for 5 min followed by discarding the supernatant and again adding PBS. Table 1 contains the list of bacteria used in this study. For the time course experiment the optical density (OD_600nm_) was adjusted so that the suspension contained 2 × 10^7^ colony forming units (CFU) mL^−1^. To explore the influence of bacterial load on plant responses, the bacterial suspensions were adjusted to 10^5^, 10^6^, 10^7^, and 10^8^ CFU mL^−1^. Next, 200 μL (time course experiment) or 1 mL (bacterial load experiment) of bacterial solution was sprayed per plant tissue culture box using an airbrush spray gun (0.2 mm nozzle diameter, Pro Dual Action 3 #83406). To obtain a homogeneous coverage, the distance between the airbrush spray gun and the plants was increased by stacking a plant tissue culture box, with the bottom cut off, onto the plant tissue culture box containing the plants being spray inoculated.

**TABLE 1:** List of bacterial strains used in this study.

Bacterial strain	References	Abbreviation
*Pseudomonas syringae* DC3000	Cuppels (1986)	Pst
*Sphingomonas* sp. Leaf34	Bai et al. (2015)	Sphingo34
*Acidovorax* sp. Leaf84	Bai et al. (2015)	Acido84
*Microbacterium* sp. Leaf347	Bai et al. (2015)	Micro347
*Williamsia* sp. Leaf354	Bai et al. (2015)	Willi354
*Pedobacter* sp. Leaf194	Bai et al. (2015)	Pedo194

### 2.3 Bacterial enumeration

Aboveground plant parts were detached from belowground parts using sterilized equipment and placed individually into preweighed 1.5 mL tubes. After determining the plant weight, 1 mL PBS with 0.02% Silwet L‐77 (Helena Chemical Company) was added to each tube. Bacteria were dislodged from the sample by shaking twice at 2.6 m s^−1^ for 5 min (Omni Bead Ruptor 24) and sonicated for 5 min. Bacterial CFU were enumerated using plate counting on R2A media plates.

### 2.4 Gene expression analysis

Four weeks old and six weeks old plants were spray inoculated with individual strains (Table 1) or PBS (mock control) for the time course and bacterial load experiment, respectively. For the time course experiment, aboveground plant parts were harvested after 1, 3, 6, 9, 12, 24, 48, and 96 hpi. For the bacterial load experiment, aboveground plant parts were harvested 96 hpi. The plant material was collected in RNase‐free microcentrifuge tubes (MCT‐150‐C, Axygen, Corning, USA) and was then immediately flash frozen in liquid N_2_. Two plants from different growth boxes were pooled per tube to form a biological replicate. Three biological replicates were sampled per treatment and time point. Flash‐frozen samples were ground to a fine powder in the collection tube using Teflon pestles (General Lab Supply, Lab Supply, Dunedin, New Zealand). RNA extraction was performed using the Isolate II RNA Plant kit (Bioline, London, England).

### 2.5 RT‐qPCR analysis

For cDNA synthesis, 1 μg of RNA was used and for the no reverse transcriptase (noRT) control, using the VitaScript First strand cDNA synthesis kit (Procomcure Biotech, Thalgau, Austria). RT‐qPCR was performed using the 2× ProPlant SYBR Mix (Procomcure Biotech) in 15 μL reaction volumes with 0.2 μM of each primer and 0.001 g L^−1^ of initial RNA in the cDNA mix. qPCRs were run using the recommended protocol for 2× ProPlant SYBR Mix (Procomcure Biotech) on a Rotor‐Gene Q (Qiagen, Hilden, Germany). Technical triplicates were performed for each sample. The ROX dye, present in the 2× ProPlant SYBR mix, was used to normalize for master mix variation between tubes. A mix of equal amounts of all cDNAs was used for normalization between runs. mRNA concentrations were calculated using Equation (1).

(1) $$\text{mRNA concerntration}\;\left(a.u\right)=1/{\left(\text{primer efficiency}\right)}^{\mathrm{Cq}}$$

Primers that were first used in this study were designed using “primer‐blast” (NCBI, Bethesda, MD, USA). Primer efficiencies were determined via serial template dilutions (Nolan et al., 2013). The mRNA concentration of each target gene was then normalized against the mean mRNA concentration of two stably expressed, previously described reference genes (Table 2; Czechowski et al., 2005). Next, the normalized mRNA concentration of each treatment (bacterial inoculation) was normalized against the mean normalized mRNA concentration of mock‐treated samples to emphasize treatment‐related changes in gene expression (Denoux et al., 2008).

**TABLE 2:** List of primers used in this study.

Template	References	5′ Primer (forward)	3′ Primer (reverse)
*ARL2*	Miebach et al. (2020)	cgaaccgtccgtacatacataa	ttgcacgaaactaaaactaaaagc
*PR1*	Miebach et al. (2020)	gatgtgccaaagtgaggtgtaa	ttcacataattcccacgagga
*At3g50280*	This work	ccttcgctggtcgtcttaac	cagagccatcaggtcgaaga
*VSP1*	Dombrecht et al. (2007)	ggacttgccctaaagaacga	gtgttctcggtcccatatcc
*LOX2*	Vogel et al. (2016)	agtcttcacgccaggctatg	gagtcctcaaccaatgggaa
*ERF1*	Vogel et al. (2016)	agttcaagagtcgctttcgg	tcgagtgtttcctcttcaacg
*PAD3*	La Camera et al. (2011)	tgctcccaagacagacaatg	gttttggatcacgacccatc
*CRK5*	Vogel et al. (2016)	tcaccactagacctcgtggatt	gtaagcatttggacgatcgc
*CRK23*	Vogel et al. (2016)	gctactccagtggattccga	aaaggcgacacagttatggc
*AT1G51890*	Vogel et al. (2016)	tgcttgagaccgctcattta	aagctgggaaacaagaatgc
*SWEET4*	Chen et al. (2010)	ccatcatgagtaaggtgatcaaga	caaaatgaaaaggtcgaacttaataagtg
*SWEET12*	Chen et al. (2010)	aaagctgatatctttcttactacttcgaa	cttacaaatcctatagaacgttggcac
*STP13*	Yamada et al. (2016)	tgtttctgtttaactccttgagaga	ttcgccgaagtcgcaaatcctcctc
*At2g28390* (reference)	Miebach et al. (2020)	ggattttcagctactcttcaagcta	tcctgccttgactaagttgaca
*At4g26410* (reference)	Miebach et al. (2020)	cgtccacaaagctgaatgtg	cgaagtcatggaagccactt

### 2.6 RNA sequencing

RNA library preparation and sequencing was performed by Custom Science (Epping, Australia). RNA quality and purity were assessed using a Nanodrop (ND‐2000 Spectrophotometer, Thermo Fisher Scientific, Waltham, MA, USA). RNA integrity was verified on a 2100 Bioanalyzer (Agilent, Santa Clara, CA, USA) using the RNA 6000 Nano Kit (Agilent). Poly‐A‐enriched libraries were prepared using the NEBNext UltraTM RNA Library Prep Kit (New England Biolabs, Ipswich, USA) for Illumina. RNA sequencing was performed on a NovaSeq 6000 (Illumina, San Diego, CA, USA) platform. Approximately 30,000,000 paired end reads with a length of 150 bp were generated per sample. Adapters and low‐quality reads (end sequences with base quality <20 and sequences with N content >10%) were removed from raw sequences and sequences below 75 bp were filtered using cutadapt (v1.9.1) (Martin, 2011). The resulting reads were mapped against the *A. thaliana* (*Arabidopsis*) reference genome (TAIR10). Mapped reads were counted with featureCounts (v1.22.2) (Liao et al., 2014).

### 2.7 RNA sequencing data analysis

Genes above 0.5 counts per million in at least three samples were used for differential gene expression analysis using edgeR (Robinson et al., 2010). Counts were scaled to effective library sizes by the trimmed mean of *M* values method (Robinson & Oshlack, 2010). Genewise dispersions were estimated via Cox–Reid profile‐adjusted likelihood and squeezed to trended dispersions using an empirical Bayes method (McCarthy et al., 2012). Genes were determined as differentially expressed using the TREAT method under edgeRs general linear model framework (McCarthy & Smyth, 2009). Genes with a fold change (FC) significantly above log_2_(1.3) and below a false discovery rate (FDR; Benjamini–Hochberg *p* correction) cut‐off of 5% were kept as differentially expressed genes (DEGs). The FC threshold was determined using elbow plots (Figures S3a and S4a). MDS plots were generated from trimmed mean of *M* values method normalized gene counts. *K*‐means clusters were calculated from log_2_‐transformed counts per million that were centered around the mean for each gene. A prior count of two was added to each gene count to prevent taking the logarithm of zero. Elbow plots were used to determine the optimal number of *K*s (Figures S3b and S4b). Heatmaps were generated from log_2_‐transformed counts per million (cpm) that were centered around the mean for each gene. GO term enrichment analysis was performed using the PANTHER classification system (v17.0) (Mi et al., 2021).

### 2.8 Chromatin state analysis

Chromatin state coordinates were obtained from Sequeira‐Mendes et al. (2014) and gene coordinates were obtained from the TAIR10 annotation from BioMart, Plantsmart28 (Durinck et al., 2005). A gene was considered to be in a certain state if at least 150 bp (approximate length of DNA wrapped around one nucleosome) of its gene body overlapped with the respective state. Thus, a single gene may have several distinct states along its coding sequence. Genes induced or repressed after Pst exposure were divided into two sets depending whether they were also differentially expressed by exposure to Micro347 or Willi354 (non‐Pst specific) or only by Pst (Pst specific). Similarly, genes induced or repressed by the highest inoculation density of Willi354 were divided into two sets depending whether they were also differentially expressed by exposure to lower bacterial densities (non‐10^8^ specific) or not (10^8^ specific). The proportion of genes of interest in each chromatin state was compared with the proportion of genes in the respective state in the complete genome. The significance of the difference between the two proportions was tested using the Marascuilo procedure, with a confidence level of 0.95.

## 3 RESULTS

**FIGURE 1:**
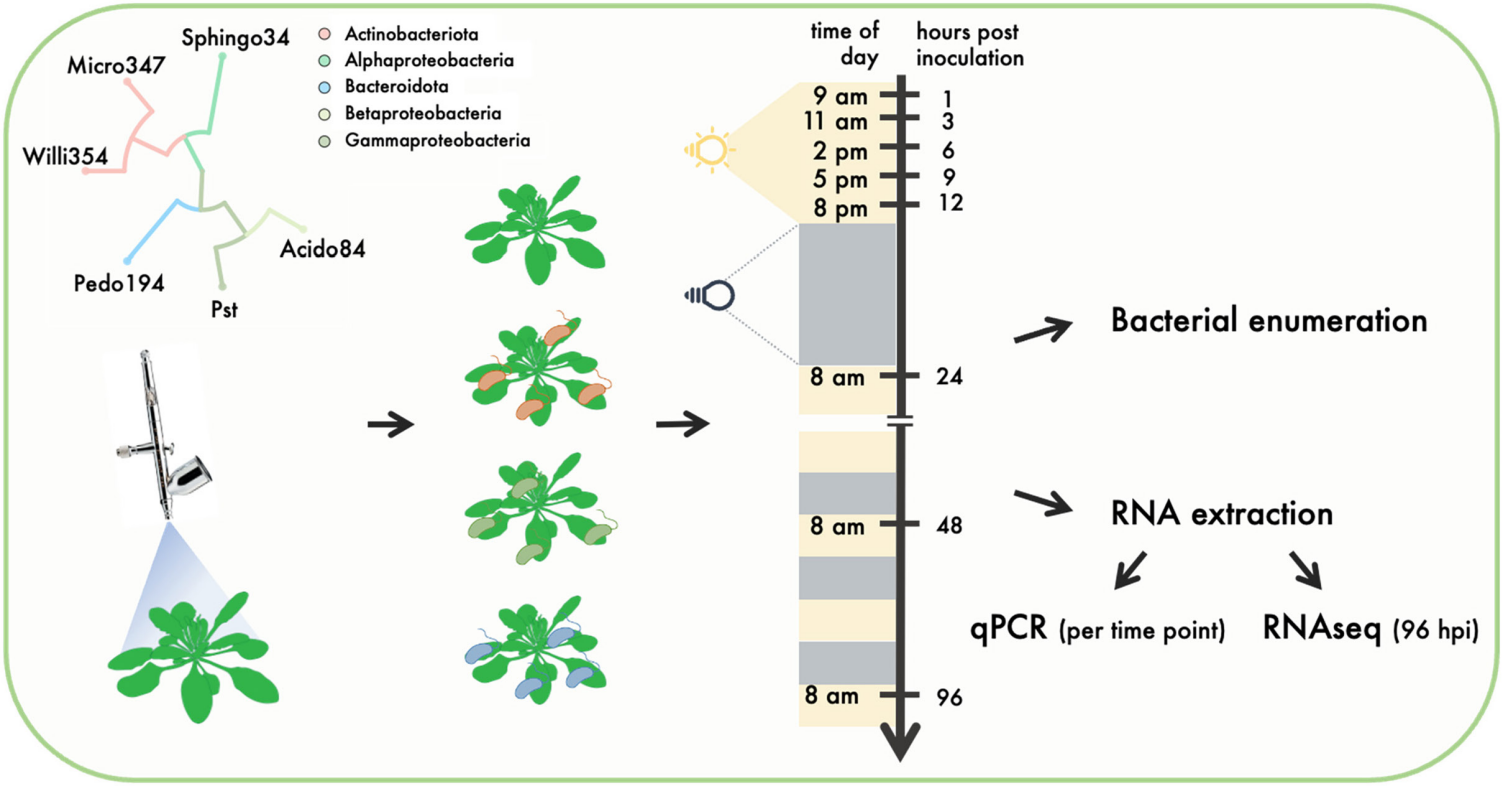
Experimental design. Four weeks old axenically grown *Arabidopsis thaliana* plants were spray inoculated with individual bacterial strains, depicted in the phylogenetic tree in the top left corner of the figure. Plants were harvested at different times after inoculation. Some plants were used for bacterial enumeration and others for RNA extraction.

The aim of this study was to broaden our knowledge of the intricate relationship between the plant and its bacterial colonizers. The focus lay on assessing plant transcriptional responses to a diverse array of microbial colonizers. Therefore, six microbial leaf colonizers representing all major phyla of the core leaf microbiota were selected (Bai et al., 2015; Vorholt, 2012) (Figure 1).

### 3.1 Temporal responses of the plant immune system

**FIGURE 2:**
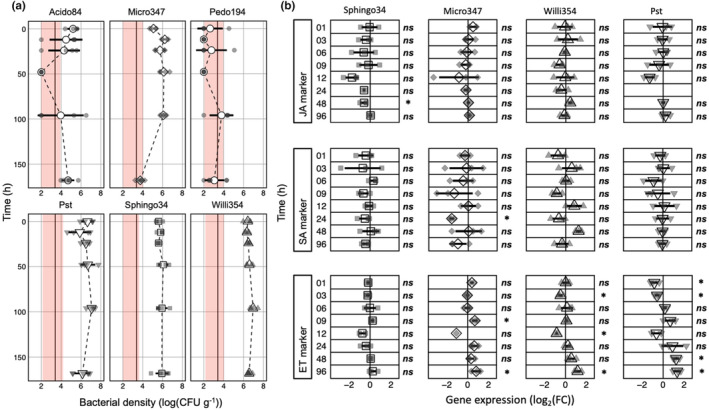
Temporal transcriptional responses to bacterial colonization. Four weeks old axenically grown *Arabidopsis* plants were spray inoculated with individual bacterial strains (see colored boxes; strains are sorted alphabetically in [a] and by response amplitude of the ethylene marker gene in [b]). (a) Bacterial density on aboveground plant parts at several times post inoculation. To accommodate plants with CFU below the limit of detection, 10^2^ CFU g^−1^ were added to the count of every sample. (b) Depicted are log_2_(FC) in gene expression relative to mock‐treated control for a jasmonic acid (JA) marker gene (*At3g50280*), a salicylic acid (SA) marker gene (*PR1*), and an ethylene (ET) marker gene (*ARL2*). Large white shapes depict the mean, bars depict standard deviation, smaller gray shapes depict individual biological replicates, dashed black line connects the means, solid black line represents the lower limit of detection based on mean plant weight, and the red areas represent the range of the limit of detection based on the heaviest and lightest plant; * depicts statistical significance (Bonferroni corrected *p* < .05) and *ns* depicts the lack of statistical significance, *T* test.

*Arabidopsis* plants were grown axenically in the “Litterbox” system, to ensure (1) low artificial, but strictly controlled, growth conditions and (2) to prevent a strong inoculation of the growth media post inoculation (Miebach et al., 2020). Four weeks old plants were spray inoculated with individual strains of bacterial leaf colonizers at ~10^5^ to 10^6^ bacteria per gram of leaf, the bacterial carrying capacity of plants in temperate environments (Burch et al., 2016; Gekenidis et al., 2017; Kniskern et al., 2007; Rastogi et al., 2012; Reisberg et al., 2012). The temporal course of bacterial densities post inoculation confirmed that bacteria were sprayed close to carrying capacity. Within 4 dpi the bacterial densities for Sphingo34 and Micro347 remained stable at 10^6^ bacteria per gram of leaf. In contrast, the bacterial densities for Willi354 and Pst slightly rose to 10^7^ bacteria per gram of leaf within 4 dpi (Figure 2a). Interestingly, the bacterial density of Micro347 significantly (*p* < .001, Tukey's HSD test) dropped to ~10^4^ bacteria per gram of leaf 7 dpi. This two‐magnitude drop in bacterial density cannot be explained by the increase in plant weight (Figure S1) and, therefore, suggests that the bacteria were dying.

Two of the six bacterial leaf colonizers, Acido84 and Pedo194, failed to consistently establish densities above the threshold of detection. Acido84 was recovered from some, but not all plants. Whenever Acido84 was recovered it reached densities of ~10^5^ to 10^6^ CFU g^−1^. This heterogeneity in colonization success was unlikely to have been caused by nonhomogeneous spray inoculation, as other inoculants exhibited considerably lower plant to plant variation (Figure 2a). Furthermore, all plants sampled 168 hpi harbored ~10^4^ to 10^6^ CFU g^−1^.

Plants inoculated by one of the four successful colonizer strains, Micro347, Pst, Sphingo34, and Willi354, were investigated further by qPCR (Figure 2b). Early temporal changes in the plant immune response were tracked using previously reported marker genes that follow the levels of the three major phytohormones in plant immunity: ET, JA, and SA (Kim et al., 2014). Marker gene expression was measured at eight different time points ranging from 1 to 96 hpi. As dynamic changes in expression were expected early after inoculation, five of the eight measurement times fell within the first 12 hpi.

Surprisingly, gene expression changes caused by the bacterial treatments were relatively weak. The strongest changes did not exceed fivefold (maximum mean: 2.5‐fold; maximum individual replicate: 4.6‐fold) in gene expression, relative to the mock‐treated control (Figure 2b). The strongest changes were observed in the expression of the ET marker, *ARL2*. Early after inoculation, its expression dropped significantly, at 1 and 3 hpi for Pst and 3 hpi for Willi354. After recovering to the expression levels found in mock‐treated plants, the relative expression of the ET marker dropped again at 12 hpi, which was significant in the case of Willi354, with Pst seemingly following the same trend. Expression levels then rose above mock‐treated control by ~2.5‐fold at 96 hpi, with changes being statistically significant for 48 and 96 hpi for Pst and 96 hpi for Willi354 (Figure 2b). In addition, Micro347 showed a significant ~1.8‐fold rise in ET marker gene expression at 96 hpi (Figure 2b).

Regarding the JA marker, statistically significant changes were only observed in response to Sphingo34. A drop in expression was observed between 12 and 48 hpi with the latter being statistically significant, but rather weak at ~1.5‐fold (Figure 2b). Expression of the SA marker, *PR1*, fluctuated strongly, but not significantly upon Willi354 treatment. Strong fluctuations between no expression change and a strong downregulation were observed following Micro347 treatment with a significant ~threefold decrease in expression at 24 hpi. Sphingo34 and Pst elicited no changes in *PR1* expression (Figure 2b).

**FIGURE 3:**
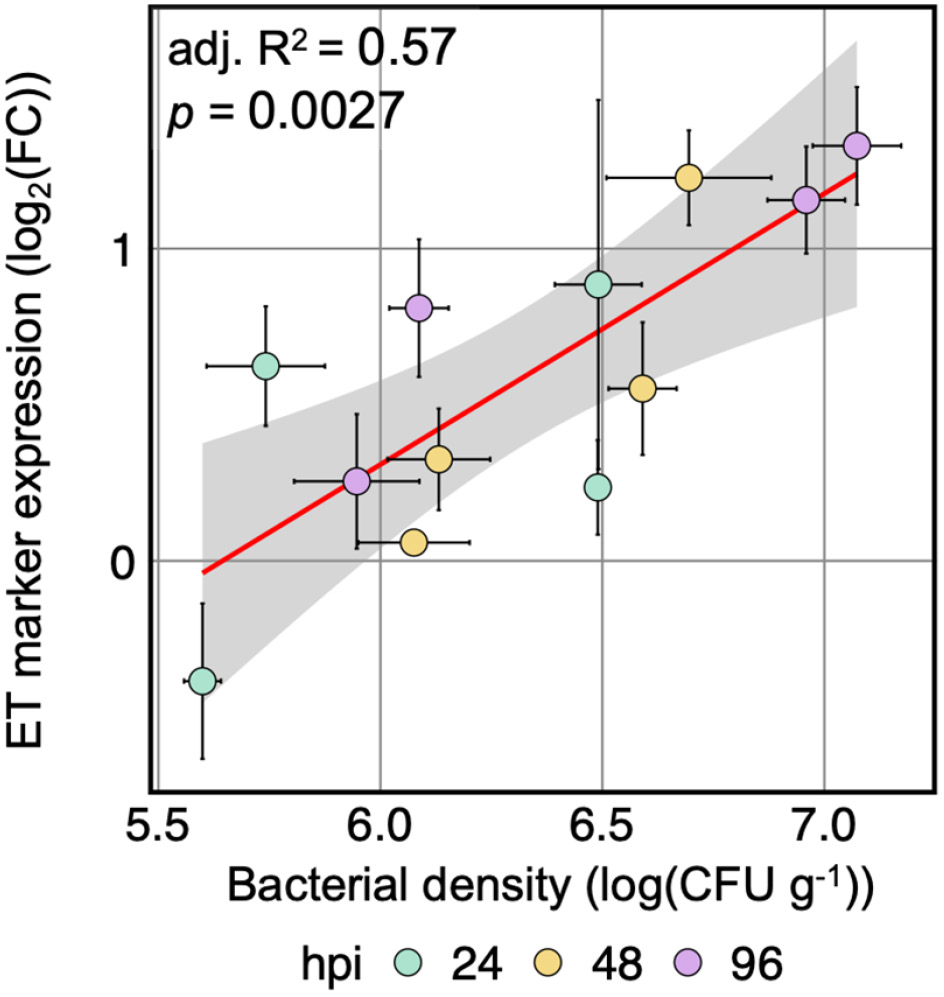
Ethylene marker responses may be explained by bacterial density. Correlation between mean log_2_(FC) in gene expression of ethylene (ET) marker gene (*ARL2*) relative to mock‐treated control and mean bacterial density. Colors depict the time of sampling post inoculation, red line depicts a fitted linear model, the gray bar depicts limits of 95% confidence interval, and error bars depict standard error of the mean. *n* = 3 and 4 for mean log_2_(FC) in gene expression and mean bacterial density, respectively.

The increase in ET marker expression within the last three sampling times (24, 48, 96 hpi) seemed to follow the increase in bacterial density of the inoculants (Figure 2a,b). Interestingly, 57% (adj. *R* ^2^ = .57, *p* = .0027) of the change in gene expression relative to the mock control can be explained by the bacterial density, irrespective of the inoculant (Figure 3).

### 3.2 Genome‐wide transcriptional changes indicate strong similarity in the plant response to various bacterial leaf colonizers

**FIGURE 4:**
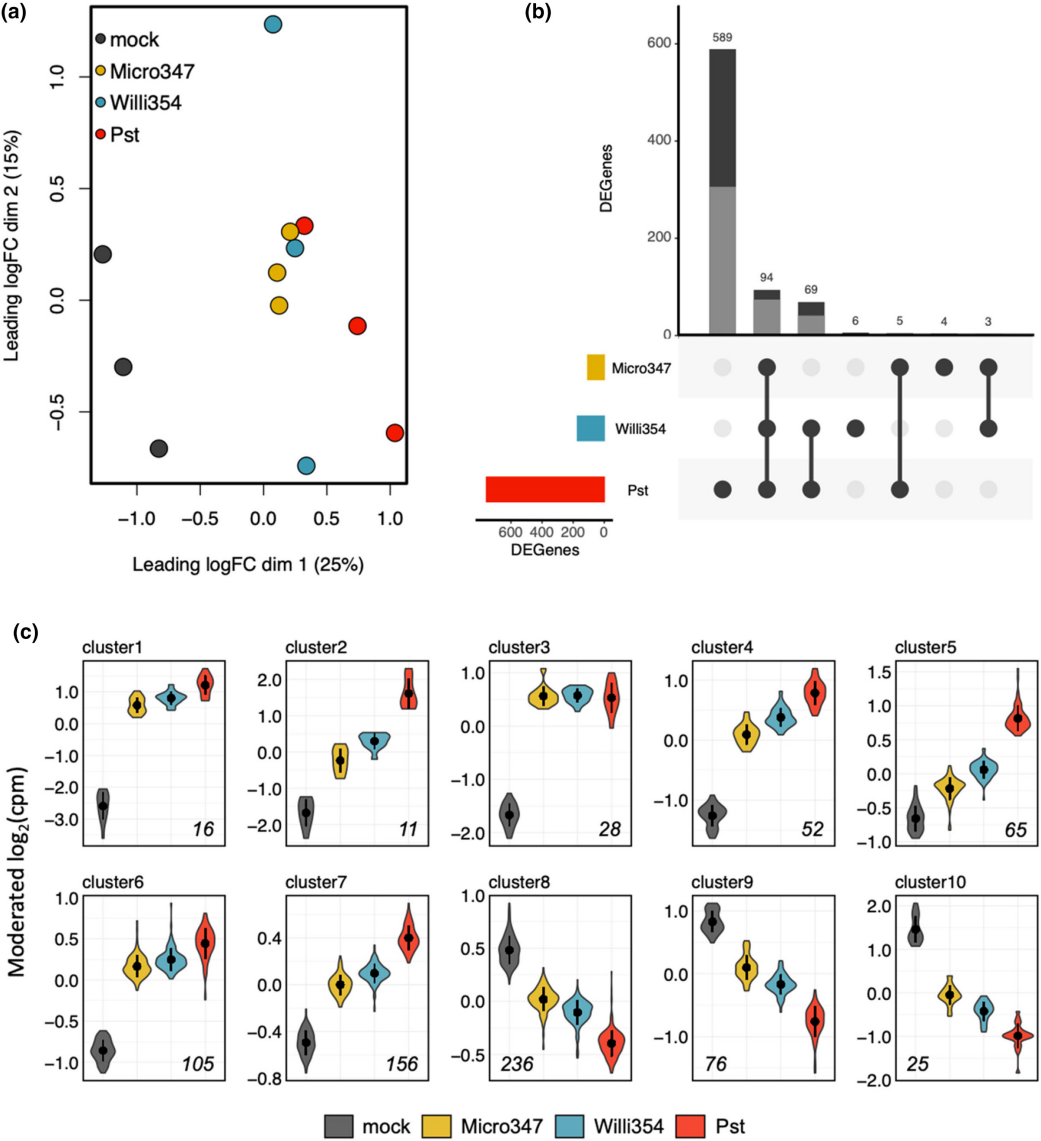
Comparison of transcriptomic response to various leaf‐colonizing strains. (a) Multidimensional scaling plot of transcriptomes of aboveground plant parts of 4 weeks old axenically grown *Arabidopsis* plants 4 days after spray inoculation with individual bacterial strains. Points depict individual transcriptomes and color depicts inoculant or mock control treatment. (b) UpSet plot of DEGs in the different treatments. The colored bar chart on the left depicts the total number of DEGs per treatment. The black dots in the panel's matrix depict unique (individual dots) and overlapping (connected dots) DEGs. The top bar chart depicts DEGs for each unique or overlapping combination in the panel's matrix. Gray bar depicts upregulated DEGs and black bar depicts downregulated DEGs. (c) Plots showing moderated log_2_(cpm) of DEGs per *k*‐means cluster of transcriptomes of aboveground plant parts of 4 weeks old axenically grown *Arabidopsis* plants 4 days after spray inoculation with individual bacterial strains. Colors depict the treatment and the italicized number in each plot depicts the number of DEGs per cluster. Treatments were ordered based on the number of DEGs. Note that the *y*‐axis differs between different cluster plots.

The strongest changes in gene expression were observed in Pst‐, Willi354‐, and Micro347‐treated plants at 96 hpi. To gain an understanding of genome‐wide transcriptional changes to individual leaf‐colonizing strains, the RNA of plants sampled at 96 hpi treated with either Pst, Willi354, or Micro347 were further subjected to RNA sequencing. The expression profiles of various target genes quantified by RNA sequencing were similar to those quantified by RT‐qPCR confirming that RNA sequencing was performed correctly (Figure S2). As expected, the transcriptomes of mock‐treated plants were distinct from those of inoculated plants, as shown by multidimensional scaling (MDS) and *k*‐means clustering (Figure 4a; Figure S3c). The transcriptomes separate by treatment along the first dimension of the MDS plot (Figure 4a). Mock and Pst‐treated samples separate the furthest, corresponding to a leading FC of ~fourfold between the two treatments. Micro347‐ and Willi354‐treated samples cluster close to Pst with Willi354 being closer to Pst than Micro347, indicating a higher overlap of differentially expressed genes (DEGs). The second dimension of the MDS plot mainly separates the individual transcriptomes within a treatment group, showing low variation within Micro347 samples and strong variation within Willi354 samples, corresponding to a leading FC of ~threefold (Figure 4a).

Genes with a significant FC threshold of log_2_(1.3) based on the edgeR TREAT algorithm (McCarthy & Smyth, 2009) and a FDR <.05 were defined as DEGs. This FC threshold was chosen, based on the median “elbow,” the point of maximum curvature (second derivative) of the number of DEGs as a function of the log_2_(FC) threshold per treatment (Figure S3a). Pst‐treated plants exhibited 757 DEGs at this FC threshold, followed by Willi354 with 172 DEGs and Micro347 with 106 DEGs. Interestingly, almost all the DEGs in Micro347 are also differentially expressed in Willi354 and Pst. In addition, almost all the DEGs found in Willi354 are also differentially expressed in Pst (Figure 4b). This shows that plant responses to these three leaf‐colonizing strains are largely similar but differ in their strength depending on the leaf colonizer. A closer look at individual gene expression changes further highlights the similarity in the responses. Most changes follow a sequence with either increasing or decreasing expression from mock‐treated control over Micro347 and Willi354 to Pst‐treated plants (Figure 4c; Figure S3c). Furthermore, all the 770 genes that were differentially expressed in any of the treatments were either up‐ or downregulated in all the treatments. No gene was significantly upregulated in one treatment and significantly downregulated in another.

To gain a better resolution of gene expression changes, the 770 genes that were differentially expressed in any of the treatments were further separated by *k*‐means clustering. *K*‐means clustering was performed based on moderated log_2_(cpm). Ten *k*‐means were chosen, based on the “elbow” of the total sum of squares as a function of the number of *k*‐means (Figure 4c; Figure S3b,c). The 433 upregulated genes are in clusters 1–7, and the 337 downregulated genes are in clusters 8–10 (Figure 4c; Figure S3c). In addition to more genes being significantly upregulated than downregulated, FCs were greater in upregulated genes. Clusters 1 and 2 contain genes with the strongest upregulation and cluster 10 genes with the strongest downregulation at FCs in moderated log_2_(cpm) of ~16‐fold and ~6‐fold, respectively (Figure 4c).

### 3.3 Transcriptional responses depend on bacterial load

As seen before, responses to bacterial colonization seem to be largely similar (Figure 4b,c) in response to the tested strains. This was especially surprising as Pst is an *Arabidopsis* pathogen, whereas Micro347 and Willi354 were isolated from leaves of asymptomatic plants (Bai et al., 2015; Cuppels, 1986). In addition, bacterial density, irrespective of the bacterial colonizer, had a highly significant effect on ethylene responses (Figure 3). Taken together, this suggests that pathogenicity is to some extent dependent on bacterial density, following Paracelsus' theory “the dose makes the poison” (Paracelsus, 1538). This raises the question whether nonpathogenicity of bacteria is merely a case of the plant balancing their proliferation or the bacteria doing so in order to avoid being penalized by the plant. To gain a better understanding of plant responses to nonpathogenic leaf‐colonizing bacteria and to determine whether the bacterial load changes the nature of the response, plants were inoculated with different concentrations of Willi354 followed by RNA sequencing. Willi354 was chosen for this experiment as it exhibited stronger responses than Micro347 in the previous experiment (Figure 4b,c).

**FIGURE 5:**
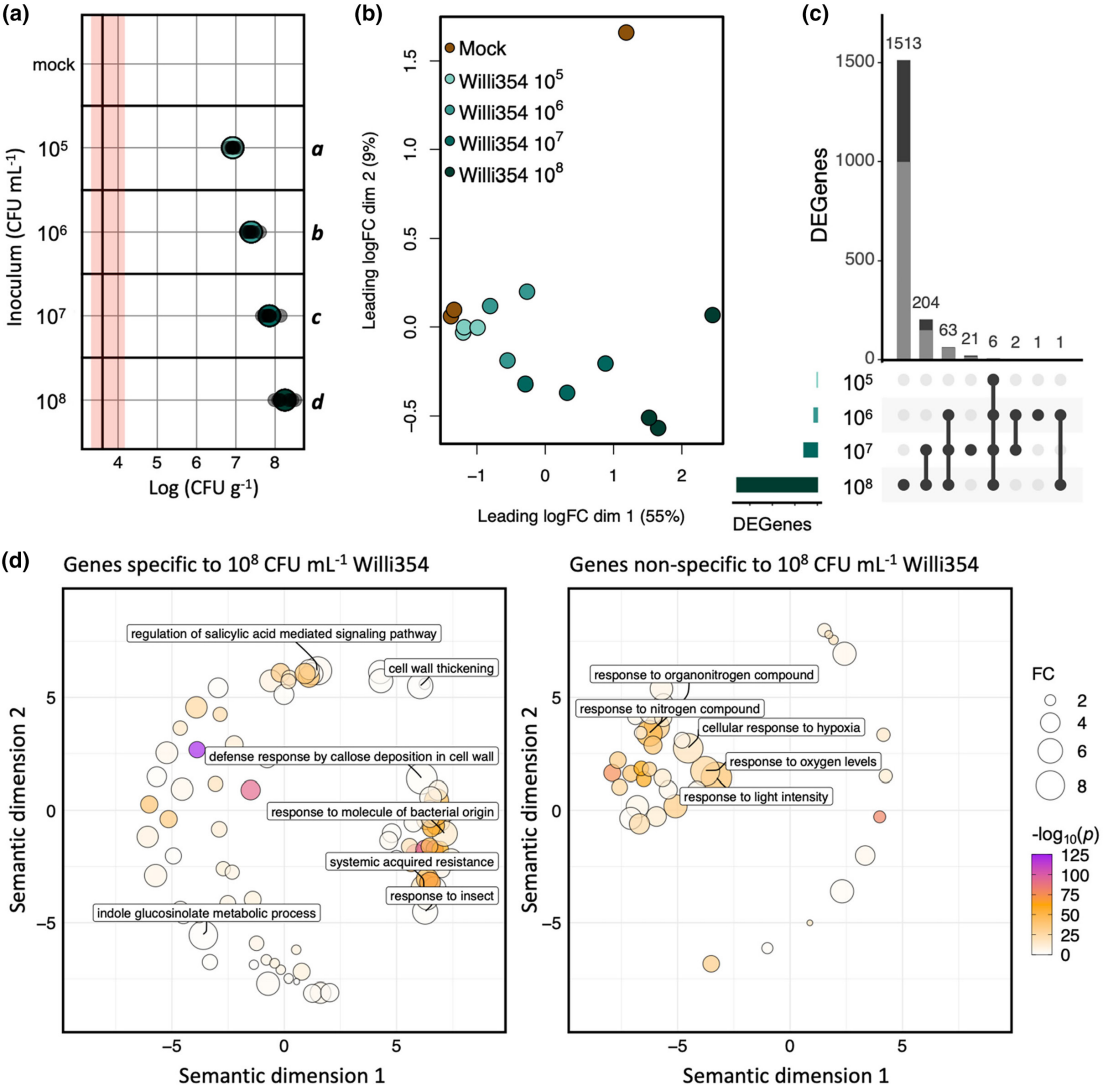
Plant transcriptomic response to different densities of Willi354. (a) Bacterial density of Willi354 on aboveground plant parts of 6 weeks old axenically grown *Arabidopsis* plants 96 hpi with different initial densities. Colored circles depict the mean, bars depict standard deviation, smaller gray circles depict the bacterial density from individual biological replicates, the vertical black line represents the threshold of detection based on mean plant weight, and red bar represents the threshold range of detection based on the heaviest and lightest plant. Letters on the right side of the plot depict statistical differences (*p* < .001, one‐way ANOVA and Tukey's HSD test). (b) Multidimensional scaling plot of transcriptomes of *Arabidopsis* aboveground plant parts 96 hpi. Circles depict individual transcriptomes and color depicts inoculation density or mock control treatment. (c) UpSet plot of transcriptomes depicted in (b). Genes with a FC significantly above log_2_(1.3) and a FDR <.05 were defined as DEGs. In each panel, the bottom left bar chart depicts the overall number of DEGs per treatment. The dots in the panel's matrix depict unique (individual dots) and overlapping (connected dots) DEGs. The top bar chart depicts DEGs for each unique or overlapping combination in the panel's matrix. Gray bar depicts upregulated DEGs, black bar depicts downregulated DEGs. (d) Functional enrichment of genes specifically and nonspecifically expressed to high densities of Willi354. Functional enrichment of GO terms distributed in the semantic space. Closeness in semantic space ideally reflects closeness in GO term structure. Circles depicted significantly enriched GO terms (*p* < .05, Bonferroni corrected). Circle size depicts fold enrichment and color depicts −log_10_(*p*). GO terms with a FC >6 are labeled.

Six weeks old axenically grown *Arabidopsis* plants were spray inoculated with Willi354 with inoculation densities ranging from 10^5^ CFU mL^−1^ to 10^8^ CFU mL^−1^. Four days after inoculation, the bacterial densities on the plants ranged from 6.57 × 10^6^ CFU g^−1^ to 3.22 × 10^8^ CFU g^−1^ and strongly correlated with the inoculation density (adj. *R* ^2^ = .9265, *p* = 3.594 × 10^−14^) (Figure 5a). The transcriptomic response of the plants changed gradually with increasing inoculation density, as seen in the MDS plot and *k*‐means clustering (Figure 5b; Figure S4). The transcriptomes separate along the first dimension of the MDS plot, which explains 55% of the variation between the different samples, with those of mock‐treated plants and those of plants inoculated with Willi354 at 10^8^ CFU mL^−1^ being most dissimilar at a leading FC of ~16‐fold (Figure 5b).

Genes were defined as DEGs as described earlier. Plants treated with 10^5^, 10^6^, 10^7^, and 10^8^ CFU mL^−1^ of Willi354 exhibited 6, 73, 296, and 1787 DEGs, respectively.

A total of 1811 genes were differentially expressed across all treatments, with ~68% (1230 DEGs) being upregulated (Figure 5c). Furthermore, upregulated genes exhibited stronger changes in gene expression, the strongest being ~30‐fold in moderated log_2_(cpm), compared to downregulated genes, the strongest being ~fourfold in moderated log_2_(cpm) (Figure S5). This highlights that positive expression changes are not only more prominent, but also stronger than negative expression changes. The DEGs were separated by *k*‐means clustering into 10 clusters. Clusters 1–8 contained the upregulated and clusters 9 and 10 contained the downregulated genes (Figures S4c and S5). Most genes showed no marked difference between mock‐treated plants and plants treated with Willi354 at 10^5^ and 10^6^ CFU mL^−1^. At concentrations of 10^7^ and 10^8^ CFU mL^−1^, genes in clusters 1, 3, 4, 6, and 8 were strongly upregulated, with genes at 10^8^ being upregulated twice as much compared to those at 10^7^ (Figure S5). Genes in clusters 5 and 7 appear to follow a sigmoidal curve, with no difference in gene expression between plants inoculated with Willi354 at 10^7^ and 10^8^ CFU mL^−1^ in cluster 7 (Figure S5). Genes in clusters 9 and 10 exponentially decreased in expression with increasing density of Willi354 (Figure S5).

To further explore the nature of plant responses to inoculation with Willi354, a GO term enrichment analysis was performed on two different sets of genes. The first set comprised genes that were exclusively differentially expressed in plants treated with Willi354 at 10^8^ CFU mL^−1^, whereas the second set comprised genes that were also differentially expressed at lower densities of Willi354. The functional profiles of both sets of genes were markedly different (Figure 5d). Genes differentially expressed exclusively at the highest density of Willi354, were greatly enriched for genes related to plant immunity, including perception of the biotic environment, such as “response to molecule of bacterial origin” and “response to insect,” metabolism of secondary metabolites such as “indole glucosinolate metabolic process,” local defense responses such as “cell wall thickening” and “defense response by callose deposition in cell wall,” and systemic defense responses such as “regulation of salicylic acid mediated signalling pathway” and “systemic acquired resistance” (Figure 5d). By contrast, genes that were also differentially expressed at lower densities of Willi354 were greatly enriched for genes related to plant nitrogen homeostasis, such as “response to nitrogen compound” and “response to organonitrogen compound,” plant oxygen levels, such as “response to oxygen levels” and “response to hypoxia” and the plant's “response to light intensity” (Figure 5d).

### 3.4 Transcriptional changes induced by bacteria may depend on the chromatin state of a given gene

**FIGURE 6:**
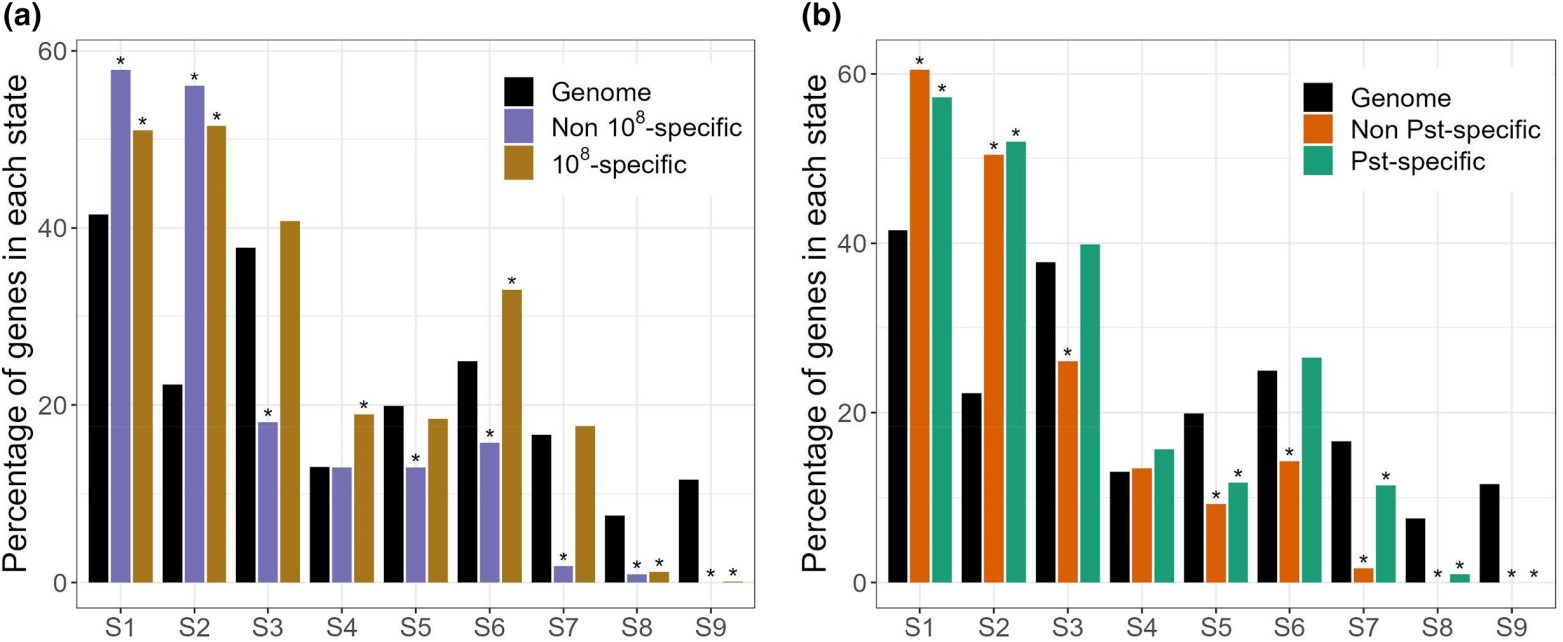
Chromatin state analysis of genes upregulated following bacterial inoculation. (a) Chromatin state analysis of genes induced either specifically by the highest density (10^8^) of Willi354 or also induced by lower inoculant densities (10^5^, 10^6^, or 10^7^). (b) Chromatin state analysis of genes induced either specifically by Pst or also induced by Willi354 or Micro347 (all at a density of 10^7^). The chromatin states coordinates were obtained from Sequeira‐Mendes et al. (2014). * indicates significant difference compared to the genome, tested by Marascuilo procedure (*α* = 0.05).

A major determinant of gene transcriptional regulation is the chromatin state of genes. Chromatin states are determined by the combination of chromatin modifications and histone variants. Various histone modifications, such as H3K4me3, H3K36me3, and lysine acetylation, were previously shown to contribute to the induction of genes in response to pathogen exposure (Berr et al., 2012; Ding & Wang, 2015). As genes specifically induced by high densities of Willi354 presented distinct functional enrichment than genes induced by Willi354 irrespective of inoculation density, we hypothesized that both sets of genes, genes activated after high inoculation and genes activated irrespectively of inoculation density, might exhibit different chromatin states prior to inoculation. To that end, the state of both sets of genes was examined using the chromatin state topology established by Sequeira‐Mendes et al. (2014) (Figure 6a). Both sets exhibited an enrichment of states 1 and 2, which are both characterized by the presence of the histone variant H2A.Z accompanied either by activating marks such as H3K4me3, H3K36me3, or by a combination of activating (H3K4me3) and repressive (H3K27me3) marks, respectively. They also displayed an underrepresentation of states 8 and 9, which contain heterochromatic marks such as H3K9me2 and H3K27me1. In addition, genes which are upregulated by Willi354 irrespective of inoculation density showed an underrepresentation of states 3, 5, 6, and 7. By contrast, genes solely induced by the highest density of Willi354 either showed an enrichment (state 6) or no difference to the reference. These results reveal that, similarly to the distinct functional enrichments, genes induced specifically by higher bacterial densities exhibit a partially different chromatin signature than genes that are also induced by lower densities.

As the genes induced solely by the higher densities of Willi354 displayed an enrichment for plant immunity and defense‐related terms (Figure 5d), we wondered whether those genes would have a similar chromatin profile as genes induced by Pst. The genes were divided into two sets, depending whether they were induced specifically by Pst or whether they were also induced by Willi354 or Micro347 (Figure 6b). Similarly to the previous analysis, both sets presented an enrichment for states 1 and 2 and an underrepresentation for states 8 and 9. They also showed an underrepresentation of state 5, characterized by the presence of the repressive mark H3K27me3. Additionally, genes induced by both Pst and Willi354/Micro347 displayed an underrepresentation of states 6 and 7, which was not observed or of reduced magnitude for the genes induced specifically by Pst. Despite limited overlap between the two transcriptomic experiments (Figure S6), the chromatin profiles of genes induced specifically either by high Willi354 densities or Pst were comparable and distinct from the profiles of genes induced also by nonpathogenic bacteria or by lower bacterial densities. This observation supports the idea that inoculation with higher densities of nonpathogenic bacteria leads to a transcriptomic response similar to that triggered by pathogenic bacteria.

### 3.5 High densities of Willi354 caused slight disease phenotypes

**FIGURE 7:**
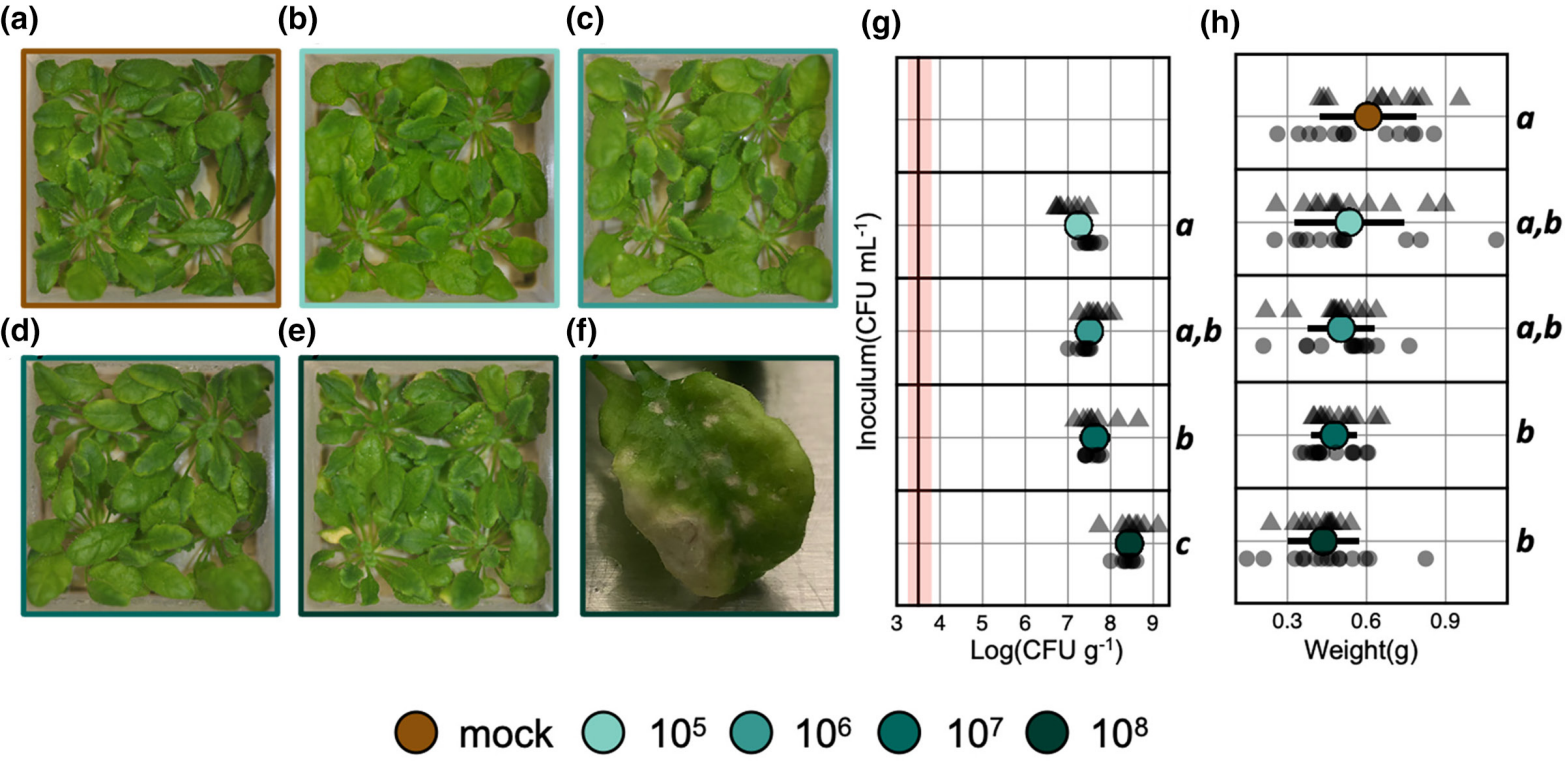
Effect of Willi354 density on plant phenotype 21 dpi. Representative images of 6 weeks old axenically grown *Arabidopsis* plants. Plants were either mock treated (a) or inoculated with Willi354 at 10^5^ CFU mL^−1^ (b), 10^6^ CFU mL^−1^ (c), 10^7^ CFU mL^−1^ (d), or 10^8^ CFU mL^−1^ (e). (f) Representative image of the leaves that showed spots of localized cell death in plants inoculated with Willi354 at 10^8^ CFU mL^−1^. (g) Bacterial density of Willi354 on aboveground plant parts of 6 weeks old axenically grown *Arabidopsis* plants. The vertical black line represents the threshold of detection based on mean plant weight and the red bar represents the threshold range of detection based on the heaviest and lightest plant. (h) Fresh weight of 6 weeks old axenically grown *Arabidopsis* plants. Colored circles depict the mean, bars depict standard deviation, and smaller gray shapes depict the bacterial density from individual biological replicates of two independent experiments. Letters on the right side of the plots depict statistical differences (*p* < .05), one‐way ANOVA, and Tukey's HSD test.

Since the genes that were uniquely differentially expressed in plants inoculated with Willi354 at 10^8^ CFU mL^−1^ were enriched for plant immunity‐related genes, plants were inoculated with Willi354 under the previous experimental conditions and sampled at 14 and 21 dpi, to investigate if Willi354 evokes plant disease phenotypes. Indeed, plants inoculated with Willi354 at 10^8^ CFU mL^−1^, but not at lower densities, exhibited necrotic lesions on a few leaves 14 and 21 dpi (Figure 7a–f; Figure S7). In addition, plant weight was negatively correlated with inoculation density of Willi354 (adj. *R* ^2^ = .11, *p* = 6.48 × 10^−5^) (Figure 7h).

## 4 DISCUSSION

### 4.1 Temporal responses of the plant immune system

Bacteria were spray inoculated at densities that matched the bacterial carrying capacity of leaves in temperate environments (Burch et al., 2016; Gekenidis et al., 2017; Kniskern et al., 2007; Rastogi et al., 2012; Reisberg et al., 2012). Four of the six inoculated bacteria successfully established on plant leaves, whereas two, Acido84 and Pedo194, failed to consistently reach bacterial densities above the threshold of detection, which was on average ~2500 CFU g^−1^ of leaf fresh weight (Figure 2a). This was rather surprising as both genera were previously found to make up more than 1% of the total bacterial population on *Arabidopsis* (Vorholt, 2012). Furthermore, both strains were recently shown to successfully colonize *Arabidopsis* (Vogel et al., 2021). However, in the study by Vogel and colleagues, colonization density was measured 9 days after drop inoculation on seedlings in an agar‐based system. In this study, the “Litterbox” system was employed, which reliably mimics environmental population densities, as opposed to agar‐based systems that exhibit unnaturally high population densities (Miebach et al., 2020). In addition, Acido84 reached population densities of 10^4^–10^6^ CFU g^−1^ in all sampled plants at 168 hpi (i.e., 7 dpi). Since Acido84 was also successfully recovered straight after inoculation, this indicates (1) that Acido84 was not harmed during the spraying procedure and (2) that it was able to thrive on leaves at later time points. Overall, this suggests that Acido84 had to acclimatize to its new environment after growth on R2A media, even though R2A is, like the phyllosphere, oligotrophic. Pedo194, in contrast, was only successfully recovered from one plant immediately after spray inoculation at a density ~2 magnitudes lower than the inoculum, suggesting that the inoculation procedure might have been detrimental to it.

Pst and Will354 rose in population size to ~10^7^ CFU g^−1^ at 96 hpi. Population size then declined to 10^6^ CFU g^−1^ at 168 hpi (Figure 2a). Whether this was due to exhaustion of resources or plant immune responses remains to be determined. Interestingly though, the expression of the ET marker *ARL2* could be explained in large parts by the bacterial density of the colonizer, irrespective of the inoculant. This suggests that *ARL2* expression must be either triggered by a common MAMP, shared between the isolates, or was triggered by many MAMPs which the plant did not distinguish between. Furthermore, it suggests that *ARL2* expression is proportional to the MAMP titer. This agrees with previous findings, which described stronger transcriptional responses to both higher pathogen and higher MAMP titers (Denoux et al., 2008; Thilmony et al., 2006).

The observed expression changes upon bacterial treatment were overall rather weak (Figure 2b). No significant expression changes were observed for either the JA or the SA marker, with the exception of a significant but weak drop in expression of the JA marker 48 h after inoculation with Sphingo34 and a significant drop in expression of the SA marker 24 h after inoculation with Micro347. It is thus inconclusive whether the JA SA antagonism is at play (Zhang et al., 2020). The strongest changes in gene expression were observed in the ET marker and culminated at 96 hpi. As expected, the strongest changes were observed in the plants treated with Pst. The overall weak and rather late response seemingly disagrees with previous studies describing fast and substantial changes in gene expression upon MAMP treatment and infection with Pst (Bjornson et al., 2021; Denoux et al., 2008; Thilmony et al., 2006; Zipfel et al., 2006). However, in these studies either young seedlings were treated by a complete change of media, with the fresh media containing the MAMP, or leaves of mature plants were vacuum infiltrated. In both cases MAMPs were readily available. By contrast, in the case of a surface spray, a sufficient amount of eliciting molecules needs to cross the hydrophobic cuticle layer to reach the plasma membranes of plant cells (Schlechter et al., 2019) or bacteria need to migrate into the apoplast (Beattie & Lindow, 1999; Melotto et al., 2006). In addition, the flg22 receptor, FLS2, is highly expressed in leaves near bacterial entry sites, such as stomata which are predominantly found on the abaxial (lower) leaf surface, and hydathodes as well as in leaf veins (Beck et al., 2014). Vacuum infiltration of bacterial suspensions would render leaf veins more exposed to MAMPs and a change in liquid media would render stomata and hydathodes more exposed to MAMPs, than in the more “natural” scenario of topical application.

### 4.2 Genome‐wide transcriptional responses to leaf colonization

#### 4.2.1 Plant responses to nonpathogenic bacteria are qualitatively similar but differ quantitatively compared to pathogenic bacteria

Bacterial colonization with nonpathogenic leaf colonizers significantly altered the expression of several genes in the host, though not to the extent of a pathogenic leaf colonizer. Remarkably, the responses observed were largely similar although weaker in response to colonization by the nonpathogenic bacteria. Most genes that were significantly expressed in response to one strain were also significantly expressed in response to strains that elicited stronger responses and, thus, had higher numbers of DEGs. None of the 770 genes with a significant FC in any of the bacterial treatments was upregulated by one strain and downregulated by another. Changes in expression of genes belonging to clusters 2, 4, 5, 6, 7, 8, 9, and 10 were either progressively increasing or decreasing when treatments were sorted by the number of DEGs that they elicited (Figure 4c). This indicates that those genes were similarly regulated in response to bacterial colonization irrespective of the symbiotic relationship of the inoculant with the plant, though less severely in response to nonpathogenic bacteria. Such similarity in the plant response to various leaf colonizers was also described recently by Maier et al. (2021), though without the context of a pathogenic bacterium. Interestingly, the response strength was strongly driven by the bacterial density of the inoculant (Maier et al., 2021), which was also observed in this study with respect to the ET marker responses to nonpathogenic and pathogenic bacteria. This suggests that plants merely responded to a pool of bacterial MAMPs quantitatively, by responding to the total amount of MAMPs present, rather than qualitatively by integrating a unique mix of different MAMPs into a tailored plant response.

#### 4.2.2 The effect of bacterial load on plant gene expression

Bacterial densities were the major driver of ethylene marker expression between 24 and 96 hpi irrespective of the bacterial colonizer (Figure 3). In addition, genome‐wide transcriptional responses to bacterial colonization were largely similar, but differed in the number of DEGs (Figure 4b), as well as in the expression strength of individual genes (Figure 4c). This was remarkable, as the tested strains included bacteria isolated from asymptomatic plants (Bai et al., 2015) as well as the pathogenic Pst (Cuppels, 1986). As pathogenicity is linked to bacterial density, this raises the question whether nonpathogenicity is merely a case of the plant limiting uncontrolled proliferation via pattern triggered immunity or bacteria limiting their proliferation to avoid being penalized by the plant.

Plants were inoculated with Willi354 at various concentrations, resulting in different bacterial densities ranging from somewhat natural densities ~10^6^ to 10^7^ CFU g^−1^ to artificially high densities ~10^8^ to 10^9^ CFU g^−1^ within 96 hpi (Figure 5a). These differences in bacterial densities were still observed at 21 dpi (Figure 7g). The maximal bacterial load, referred to as the carrying capacity, strongly correlated with the inoculation density (Figure 5a), as was previously demonstrated on bean leaves (Remus‐Emsermann et al., 2012; Wilson & Lindow, 1994). Interestingly, severe disease phenotypes were observed on a few leaves of plants colonized by Willi354 at ~10^8^ to 10^9^ CFU g^−1^ 14 and 21 dpi (Figure 7e,f; Figure S7). In addition, plant weight negatively correlated with bacterial density (Figure 7g,h). This suggests that bacteria that are otherwise nonpathogenic can be detrimental to the plant at very high densities. Whether this is caused by changes in bacterial behavior at high inoculation densities remains inconclusive. Differences in environmental cues perceived by bacteria can lead to changes in behavior and potentially explain a shift from mutualism to opportunistic pathogenicity (de Vries et al., 2020; Jochum & Stecher, 2020; Ma et al., 2021; Schulz & Boyle, 2005). Here the experiments were conducted in the litterbox system (Miebach et al., 2020), a tightly controlled environment. Thus, differences in bacterial context are limited to (1) differences in their density potentially causing changes in bacterial behavior by quorum sensing responses (Dulla & Lindow, 2008) and (2) differences in plant responses translating into changes in the phyllosphere chemistry.

RNA sequencing revealed an exponential increase in both the number of DEGs and the expression pattern of most DEGs to increasing bacterial densities (Figure 5c; Figure S5). In addition, gene expression changes were relatively strong in upregulated genes with FCs up to ~30‐fold (Figure S5). This suggests that the plant barely invests energy into an interaction with its bacterial colonizers at low bacterial densities, but markedly increases responses when bacteria are reaching potentially dangerous levels. Accordingly, genes that were uniquely differentially expressed in plants harboring Willi354 at ~10^8^ to 10^9^ CFU g^−1^ were greatly enriched for immune‐related GO terms, including perception of the biotic environment (“response to molecule of bacterial origin,” “response to insect”), metabolism of secondary metabolites (“indole glucosinolate metabolic process”), local defense responses (“cell wall thickening,” “defense response by callose deposition in cell wall”), and systemic defense responses (“regulation of salicylic acid mediated signalling pathway,” “systemic acquired resistance”) (Figure 7d). Enrichment of the latter two GO terms might suggest that, in addition to eliciting more direct defense responses, Willi354 might be able to prime the plants against future colonization of pathogens similar to previous findings (Conrath et al., 2015). Future research is needed to confirm potential priming by examining distal, noninoculated tissues.

Due to the fact that the plant “ignores” bacteria at low densities, it appears unlikely that the plant limits bacterial proliferation by active signaling processes at low bacterial densities. Consequently, it is likely that bacteria limit their proliferation in order not to alert the plant immune system. This is in agreement with recent findings showing endophytic bacteria remaining in population stasis by a multiplication–death equilibrium independent of bacterial density (Velásquez et al., 2022).

The chromatin state analysis further supported the idea that artificially high densities of nonpathogenic bacteria lead to a transcriptomic response similar to that triggered by pathogenic bacteria. Genes induced by bacterial inoculation, both at pathogenic and nonpathogenic levels, were enriched for the chromatin states 1 and 2 (Figure 6) and, specifically, were enriched in H2A.Z and H3K4me3‐H3K27me3 marks, previously shown to be essential for gene responsiveness to environmental changes (Coleman‐Derr & Zilberman, 2012; Faivre & Schubert, 2023; Sura et al., 2017). This suggests that these chromatin marks might be guiding the transcriptomic response of the plant to bacterial inoculation.

## 5 CONCLUSION

We show that plant responses to various leaf colonizing bacteria are largely similar, both in the overlap of DEGs and the expression of individual DEGs, but differ in expression strength. We tested the competing hypotheses that plants are either (1) monitoring bacterial population density or (2) differentiating between different bacterial colonizers. Our results suggest that plants are responding to bacterial densities rather than bacterial identities, favoring hypothesis (1) to be the more prevalent mode of plant response to bacteria. It appears that the plant barely invests resources into an interaction with its associated bacteria at low bacterial densities, but markedly increases gene expression toward defense responses upon high bacterial colonization.

## CONFLICT OF INTEREST STATEMENT

The authors declare that they have no conflict of interest.

## Supporting information


Data S1:

## Data Availability

The data that support the findings of this study are openly available in NCBI Gene Expression Omnibus at https://www.ncbi.nlm.nih.gov/geo/, reference number GSE232254.
